# Drug–Drug Interactions in Patients with Acute Respiratory Distress Syndrome

**DOI:** 10.3390/pharmaceutics16030303

**Published:** 2024-02-21

**Authors:** Thorsten Bischof, Christoph Schaller, Nina Buchtele, Thomas Staudinger, Roman Ullrich, Felix Kraft, Marine L. Andersson, Bernd Jilma, Christian Schoergenhofer

**Affiliations:** 1 Department of Clinical Pharmacology, Medical University of Vienna, 1090 Vienna, Austria; thorsten.bischof@meduniwien.ac.at (T.B.); bernd.jilma@meduniwien.ac.at (B.J.); 2 Intensive Care Unit 13i2, Department of Medicine 1, Medical University of Vienna, 1090 Vienna, Austria; nina.buchtele@meduniwien.ac.at (N.B.); thomas.staudinger@meduniwien.ac.at (T.S.); 3 Department of Anesthesiology, Medical University of Vienna, 1090 Vienna, Austria; roman.ullrich@meduniwien.ac.at (R.U.); felix.kraft@meduniwien.ac.at (F.K.); 4 Division of Clinical Pharmacology, Department of Laboratory Medicine, Karolinska Institutet, Karolinska University Hospital, 141 86 Stockholm, Sweden; marine.andersson@regionstockholm.se

**Keywords:** potential drug–drug interactions, intensive care unit, acute respiratory distress syndrome, extracorporeal membrane oxygenation, coronavirus disease 2019

## Abstract

Acute respiratory distress syndrome (ARDS) is a potential life-threatening, heterogenous, inflammatory lung disease. There are no data available on potential drug–drug interactions (pDDIs) in critically ill patients with ARDS. This study analyzed pDDIs in this specific cohort and aimed to investigate possible associations of coronavirus disease 2019 (COVID-19) as an underlying cause of ARDS and treatment with extracorporeal membrane oxygenation (ECMO) with the occurrence of pDDIs. This retrospective study included patients ≥18 years of age diagnosed with ARDS between January 2010 and September 2021. The Janusmed database was used for the identification of pDDIs. A total of 2694 pDDIs were identified in 189 patients with a median treatment duration of 22 days. These included 323 (12%) clinically relevant drug combinations that are best avoided, corresponding to a median rate of 0.05 per day. There was no difference in the number of pDDIs between COVID-19- and non-COVID-19-associated ARDS. In patients treated with ECMO, the rate of the most severely graded pDDIs per day was significantly higher compared with those who did not require ECMO. PDDIs occur frequently in patients with ARDS. On average, each patient may encounter at least one clinically relevant drug combination that should be avoided during their intensive care unit stay.

## 1. Introduction

Acute respiratory distress syndrome (ARDS) is a heterogenous, potentially life-threatening, inflammatory lung injury [1]. There are numerous causes, with most of them affecting the lungs (e.g., pneumonia, aspiration), while non-pulmonary causes are less frequently the trigger (e.g., sepsis, trauma) [2]. Diagnostic criteria include an acute onset within seven days, an impaired alveolar gas exchange leading to acute respiratory insufficiency, the absence of cardiac causes that may cause the condition, and bilateral opacities in chest radiographs [3].

ARDS accounts for approximately 10% of the intensive care unit (ICU) stays worldwide and is associated with a mortality rate of 35% [4]. There are specific guidelines for the non-pharmacological treatment of ARDS. However, no specific pharmacotherapy is available for this heterogenous syndrome, except for a clear recommendation to treat and/or remove any causative conditions. Therefore, an individualized treatment approach is needed for each patient [5,6]. The cornerstone of treatment remains oxygen support; more specifically, it comprises the application of protective ventilation strategies [7] and possibly the support of other failing organs. Modern ICU treatment typically requires the use of many different pharmacological agents. Therefore, patients admitted to an ICU may be particularly at risk of experiencing drug–drug interactions (DDI) [8,9]. Uijtendaal et al. reported that twice as many patients that were admitted to an ICU may have potential DDIs (pDDIs) compared to patients treated in general wards [10]. However, there are no data available on pDDIs for critically ill patients with ARDS.

The coronavirus disease 2019 (COVID-19) was the main cause of ARDS in recent years [11]. The patient population with the highest risk were elderly men with multiple chronic comorbidities [12,13]. This description implies a high number of concomitant medications and consequently a high risk of pDDIs [14]. Moreover, the specific treatment of COVID-19 may bear a relevant interaction potential, which is true for several experimental treatments that were used in the beginning of the pandemic (including antimicrobial agents, antirheumatic drugs, and corticosteroids [12,15,16,17]) as well as the later approved nirmatrelvir/ritonavir combination therapy [18]. Data are available on pDDIs in different COVID-19 settings [19,20,21], but not for COVID-19-induced ARDS in ICUs. To the best of our knowledge, this is the first study that focuses on pDDIs in critically ill patients with ARDS.

We hypothesized that pDDIs among patients with ARDS, both non-COVID-19- and COVID-19-induced, may be frequent. Our aim was to identify the most common interactions that may pose relevant risks to patients and to raise awareness for health care professionals. Furthermore, we aimed to investigate potential differences in pDDIs between COVID-19- and non-COVID-19-induced ARDS, and between patients treated with extracorporeal membrane oxygenation (ECMO) or patients who were conservatively managed. Bakker et al. pointed out two important limitations with regard to pDDI analyses that use a 24 h observation period: (i) overestimating pDDIs involving short-acting drugs and (ii) underestimating pDDIs involving long-acting drugs [22]. Therefore, in a novel approach, we (i) investigated a possible influence of the time-gap for the analysis of pDDIs (24 h vs. 1 h period) and (ii) took the estimated drug elimination time (based on published half-lives) into account.

## 2. Materials and Methods

This retrospective study analyzed medical data from patients who developed ARDS and were treated at the University Hospital of the Medical University of Vienna, the Vienna General Hospital, between January 2010 and September 2021. Prior to this study, no data were available on pDDIs for patients with ARDS, both non-COVID-19- and COVID-19-induced, rendering a formal sample size calculation impossible. Based on data from other ICU studies, a high number of pDDIs were expected. To obtain a realistic overview, we planned to include 100 patients with non-COVID-19- and 100 patients with COVID-19-induced ARDS. Furthermore, a balanced proportion of patients with and without ECMO treatment was sought.

Patients ≥18 years of age diagnosed with ARDS, both non-COVID-19- or COVID-19-associated, were eligible. The diagnosis of ARDS and COVID-19 was based on discharge letters. However, COVID-19 diagnoses were verified with polymerase chain reaction results. We included patients that were administered to the hospital until September 2021. The Janusmed interactions database, accessible at www.janusmed.se was used to identify pDDIs (accessed on 31 November 2022) [23]. Only systemically active pharmaceutical ingredients were evaluated, and the route of administration was considered. Janusmed provides information about pDDIs in four categories: clinically relevant interaction that is best avoided (D), clinically relevant interaction that can be handled (e.g., dose adjustments) (C), clinical outcome of the interaction is uncertain and/or may vary (B), and minor interaction of no consequence (A).

Demographics (e.g., age, gender) and information on pharmacological treatments were obtained from the electronic medical records of the ICUs of the Department of Anesthesiology and the Department of Medicine I and were analyzed with non-parametric descriptive statistics (e.g., median, interquartile range (IQR)). The main analysis was performed on a daily basis, considering all drugs taken within a 24 h period (i.e., from 0 to 24 h). Consequently, pDDIs were defined as the intake of two interacting medications within a 24 h period. The primary objective of this analysis was the total number and the severities of pDDIs in the overall population, and between coronavirus disease 2019-associated acute respiratory distress syndrome (CARDS) and non-CARDS, as well as between patients with and without ECMO. Moreover, the rate of pDDIs per treatment day, the most frequently involved drug classes and drug pairs, as well as the possible clinical consequences were analyzed. Between group differences in pDDIs were calculated using the Wilcoxon rank-sum test, with a two-sided α-error of 5%.

As mentioned above, the main analysis (24 h period) may overestimate the number of pDDIs involving drugs with short half-lives, while it may underestimate pDDIs associated with drugs with long half-lives [22]. To overcome these relevant limitations in the analysis of pDDIs, we conducted a second analysis in a subgroup of randomly selected 20 patients (*n* = 10 of each ARDS group) while (i) taking the elimination of the drugs into account and (ii) repeating the analysis using an hourly time frame. The respective elimination half-lives were taken from current versions of the respective Summary of Product Characteristics (SmPC). Drug elimination was approximated by multiplying the half-lives by 4, which is commonly accepted as a measure of almost total drug elimination. The electronic health records provide exact times of when a drug was administered, which is especially important for short-acting agents and the analysis using an hourly time frame. Hence, the drug elimination approach with a 1 h time frame may provide a more realistic estimate. Random selection was performed using Microsoft Excel Version 16 (Microsoft Corporation, Redmond, WA). In short, each patient received a random number. They were then ranked from smallest to largest, and the first ten patients of both cohorts (non-CARDS and CARDS) were chosen. The results of these two distinct pDDI analyses (24 h vs. 1 h period) were compared descriptively.

Drugs were categorized using Anatomical Therapeutic Chemical (ATC) codes: alimentary tract and metabolism (A), blood and blood forming organs (B), cardiovascular system (C), dermatologicals (D), genito-urinary system and sex organs (G), systemic hormonal preparations, exclusive sex hormones and insulins (H), anti-infectives for systemic use (J), antineoplastic and immunomodulating agents (L), musculoskeletal system (M), nervous system (N), antiparasitic products, insecticides and repellents (P), respiratory system (R), sensory organs (S), and various (V).

## 3. Results

This study included 200 patients diagnosed with ARDS between January 2010 and September 2021, including 100 patients with CARDS and 100 patients with non-CARDS. Eleven patients were excluded due to the unavailability of a polymerase chain reaction test result for severe acute respiratory syndrome coronavirus 2 (SARS-CoV-2) in the electronic records of the University Hospital Vienna, leaving only 89 patients in the CARDS group. Among the 189 patients, 104 required ECMO treatment (58 with COVID-19, 46 without COVID-19).

The median age of the overall population was 54 years (IQR 41–63), 64 (34%) of the patients were female, and the median treatment duration of their ICU stay was 22 days (IQR 11–36). Notably, patients with non-CARDS and ARDS were younger than patients with CARDS and ARDS. Furthermore, the ICU treatment duration of patients requiring ECMO treatment was longer.

A total of 2694 pDDIs were identified, 323 pDDIs (12%) were classified as severity D (clinically relevant interaction that is best avoided), 1214 pDDIs (45%) as severity C (clinically relevant interaction that can be handled), and 1157 (43%) as severity B (clinical outcome of the interaction is uncertain and/or may vary). These data were standardized to ICU treatment duration (per day). Overall, 0.52 pDDIs (IQR 0.37–0.83) per day were observed, while 0.05 D-graded pDDIs per day occurred in the included patients. There were no numerical differences in the occurrence of pDDIs that were normalized by treatment duration between CARDS and non-CARDS overall, and between patients with or without ECMO treatment (Table 1). 

### 3.1. Clinically Relevant Interactions That Are Best Avoided (D-Graded pDDIs)

There was no difference in the frequency of D-graded pDDIs between CARDS and non-CARDS. However, the pDDIs with the highest severity grading were significantly more common in patients with ECMO. The most frequently identified potential drug combinations that should be avoided in patients with ARDS are presented in Table 2, and the most frequently identified pDDIs across all subgroups are listed in Table S1 in Supplementary Materials. In addition, the involved organ system, whether the interaction is of a pharmacodynamic (PD) or pharmacokinetic (PK) nature, and the potential clinical consequences of these pDDIs are listed in Table 2.

In this context, the most common drug pairs by ATC codes of these p-DDIs are listed in Table 3. The drug groups of the cardiovascular system (C), anti-infectives for systemic use (J), and the nervous system (N) had the highest event rates.

### 3.2. Drug Elimination vs. 24 h Analysis

Two different analysis methods were used to identify pDDIs in a subset of 20 randomly chosen patients from both ARDS groups (*n* = 10 non-CARDS and *n* = 10 CARDS), including 12 patients that required ECMO treatment. Demographics and detailed pDDI differences of this subpopulation are presented in Tables S2 and S3. One method considered the estimated drug elimination times (expected half-life multiplied by 4) and analyzed pDDIs in a 1 h time frame. The second method was the “standard” method, which considered all drugs that were administered within a single day (0 to 24-h), regardless of their expected drug elimination time.

In the drug elimination-based analysis, a total of 220 pDDIs were identified with the following severity grades: 19 D-graded pDDIs, 96 C-graded pDDIs, and 105 B-graded pDDIs. A total of 209 pDDIs were analyzed in the 24 h analysis with the following severity grades: 17 D-graded pDDIs, 98 C-graded pDDIs, and 94 B-graded pDDIs. In eleven patients, the identified pDDIs differed between the two methodological approaches (Table 4). However, in the remaining nine patients, the number of pDDIs and drug combinations was identical. The maximum number of differences between the two analytical approaches was three, which were observed in three different patients (IDs 5, 10, 12 (Table 4)).

Table 4 presents only the drug pairs that were not identified by the respective other analytic method that was used. The pDDIs that were identified by both methods are not presented.

## 4. Discussion

This study investigated pDDIs in patients diagnosed with ARDS, both non-COVID-19- and COVID-19-associated, as well as possible associations with ECMO treatment.

In our cohort of 189 patients, we identified a total of 2694 pDDIs. Among these, 12% were categorized as clinically relevant (D) drug combinations that should be avoided. Notably, the median rate of D-graded pDDIs per day was 0.05 (0.00–0.10). Given a median ICU stay of 22 days, each ARDS patient, on average, receives at least one such drug combination. Interestingly, other ICU studies in different settings with a focus on pDDIs reported considerably lower numbers of pDDIs per patient [22,24,25,26]. Of course, such numbers are difficult to compare, given that different pDDI databases were used in different studies (i.e., G-standard drug database [22], Micromedex^®^ (Merative L.P., Ann Arbor, MI, USA) [24], www.drugs.com, accessed on 25 January 2024 (Drugsite Trust, Auckland, New Zealand) [25], Lexicomp^®^ (Wolters Kluwer, Alphen aan den Rijn, The Netherlands) [26]). It is well known that clinical decision support systems (CDSS) frequently produce varying results, both with regard to severity grading and with regard to the number of pDDIs [27,28]. The main risk factor for pDDIs is the number of different systemically active substances [29]. However, the very dynamic drug therapy in ICUs with the frequent use of short-acting agents and regular adaptions to drug therapy make an exact analysis of the number of concomitantly used drugs almost impossible; a pronounced polypharmacy in this population is the most likely cause.

Another possible explanation for a higher number of pDDIs being observed may be the choice of drugs. Infectious diseases are frequently the trigger of ARDS. Furthermore, patients who are mechanically ventilated frequently suffer from ventilator-associated pneumonias that require antimicrobial treatment. Anti-infectives are among the most high-risk drugs with regard to pDDIs. Furthermore, patients with ARDS frequently require intense analgosedation to allow a protective ventilation strategy. This may be even more essential for patients requiring ECMO therapy. Central nervous system (CNS)-active drugs are also frequently subject to pDDIs. Baniasadi et al. investigated the most common drug classes that cause the most severe pDDIs in critically ill patients in a cardiothoracic ICU. They reported that anti-infectives are the most frequently involved drugs, followed by CNS agents [8]. Another study in surgical ICUs reported an involvement of CNS drugs in 40% of all pDDIs, whereas midazolam was the most common interaction partner [30]. Finally, a third study in a tertiary care ICU also found that CNS drugs were involved in 51% of all pDDIs, followed by anti-infectives that were interaction partners in 14% of all cases [31]. In this context, this study confirms these findings because these two drug classes were also the most frequent interaction partners (Table 3). Healthcare professionals should be especially wary of pDDIs with regard to these drug classes.

In our analysis, we did not observe a difference between patients who were ECMO- and non-ECMO-treated concerning the overall occurrence of pDDIs. However, the most severely graded pDDIs were significantly more frequent in the ECMO group. We assumed that patients receiving ECMO treatment may require more analgosedation, a more intense anticoagulation, and may be sicker overall. For instance, studies reported higher infection rates among patients who were ECMO-treated compared to patients who were non-ECMO-treated [32,33,34]. Moreover, we observed a longer treatment duration in the ECMO subgroup. Along these lines, it may therefore not be surprising to find midazolam, vasopressin, and erythromycin as the most frequent interaction partners in patients requiring ECMO treatment. 

Interestingly, anticoagulation and an increased risk of bleeding was not a significant factor in our results, with an overall occurrence of eight pDDIs. Bleeding or thromboembolic events are reported as one of the most common complications in patients requiring ECMO treatment [35]. However, according to our data, these events may not be linked to pDDIs, but rather caused by the overall invasiveness of the procedure.

The novel attempt to analyze pDDIs that takes drug elimination into account and that uses an hourly time frame did not result in major differences compared to the 24 h analysis at first glance. While short-acting drugs did not contribute to the different results from the two analytical approaches, long-acting drugs, such as amiodarone, are the main reason for the observed differences. In the standard pDDI analysis method (24 h period), long-acting agents are only analyzed on the days when they are actually administered. If their elimination time is longer than 24 h, however, these drugs should also be included in the analysis of pDDIs on consecutive days. Hence, this methodological approach can be a fundamental improvement to pDDI analysis because relevant pDDIs may easily be overlooked by omitting long-acting drugs. For our analysis, we derived terminal elimination half-lives from published SmPCs and estimated drug elimination times, but we neither considered organ dysfunction nor performed therapeutic drug monitoring. Future prospective trials should overcome these obvious limitations.

Of note, ICUs are a special working environment (including continuous monitoring of vital signs, electrocardiograms), frequent laboratory analyses (including therapeutic drug monitoring), frequent assessment of sedation, and application of sedation scores and almost continuous care by nurses and physicians. Consequently, some of the most critical pDDIs may be identified immediately (e.g., QTc prolongation or arrhythmias) and may be less problematic in patients staying at an ICU compared to outpatients [36]. Special attention should be directed to antimicrobial substances because many of them induce and/or inhibit cytochrome enzymes. Pharmacokinetic interactions may occur and may further be exacerbated by organ dysfunction. However, such pDDIs might be avoidable by choosing fewer problematic alternatives. For instance, the pharmacokinetic pDDI of erythromycin–midazolam was one of the most frequent across all subgroups in our analysis. Erythromycin, commonly used in ICUs for its prokinetic effect rather than its antimicrobial activity, has the potential to prolong the QT interval and to potently inhibit CYP3A4 enzymes [37]. Azithromycin has similar prokinetic effects, while it does neither impact the QT interval nor inhibit cytochrome enzymes [38]. Other alternatives include metoclopramide or prucalopride, which have a less pronounced interaction potential [39]. Furthermore, combinations of fluconazole or voriconazole with midazolam were frequent, which may greatly prolong the CNS effects of midazolam. A study in healthy volunteers reported a 2- to 3-fold increase in the area under the midazolam plasma concentration–time curve via fluconazole due to CYP3A4 enzyme inhibition [40].

The authors emphasize that a retrospective, purely database-driven analysis of pDDIs cannot replace the expertise and an individual case-by-case assessment of a multidisciplinary team.

This study has certain limitations and biases: first, its retrospective character; second, a time bias between patients with non-CARDS and CARDS with ARDS, changes in treatment guidelines (especially an increasing use of ECMO), changing SARS-CoV-2 variants, and the approval of new drugs and vaccines. Only one database was used for this analysis. As mentioned previously, there is a relevant variability between different databases when analyzing pDDIs with regard to the number and severity of pDDIs. However, severe and clinically relevant pDDIs are usually reliably identified by all databases. This study, besides analyzing pDDIs in a patient population for which no data were available, focused on the comparison of two different methodological approaches, rather than the comparison of different databases, especially as such comparative studies have already been conducted and published by others [41,42,43]. We did not consider organ function nor perform therapeutic drug monitoring. Furthermore, data from only one tertiary care hospital were analyzed, which may limit the generalizability of this study to other populations.

## 5. Conclusions

Potential DDIs are frequent in patients with ARDS requiring intensive care treatment, regardless of COVID-19 as an underlying cause. ECMO treatment was associated with more frequent pDDIs that are clinically relevant and should be avoided. Healthcare professionals should aim to select drugs with a lower potential of causing relevant drug–drug interactions.

## Figures and Tables

**Table 1:** Demographics and pDDIs of all patients (overall) and when they were categorized into groups.

	Overall	Non-CARDS	CARDS	No ECMO (Both ARDS Conditions)	ECMO (Both ARDS Conditions)
Included patients, n	189	100	89	85	104
Sex, female (%)	64 (34)	34 (34)	30 (34)	30 (35)	34 (33)
Age, median (IQR)	54 (41–63)	49 (34–62)	58 (49–64)	56 (34–68)	54 (44–61)
ICU days, median (IQR)	22 (11–36)	21 (12–36)	23 (11–35)	16 (8–26)	26 (17–43)
pDDIs	2693	1477	1216	1000	1693
D-graded pDDIs (%)	323 (12)	193 (13)	130 (11)	107 (11)	216 (13)
C-graded pDDIs (%)	1214 (45)	648 (44)	566 (47)	471 (47)	743 (44)
B-graded pDDIs (%)	1156 (43)	636 (43)	520 (43)	422 (42)	734 (44)
pDDIs/day (median; Including B-, C- and D-graded pDDIs)	0.52 (0.37–0.81)	0.57 (0.38–0.95)	0.50 (0.35–0.75)	0.56 (0.38–0.85)	0.51 (0.37–0.74)
D-graded pDDIs/day, median	0.05 (0.00–0.10)	0.06 (0.00–0.12)	0.04 (0.00–0.09)	0.01 (0.00–0.09)	0.05 * (0.01–0.10)
C-graded pDDIs/day, median	0.22 (0.12–0.41)	0.22 (0.12–0.43)	0.20 (0.12–0.33)	0.25 (0.12–0.44)	0.21 (0.12–0.34)
B-graded pDDIs/day, median	0.24 (0.17–0.33)	0.26 * (0.18–0.36)	0.21 (0.16–0.31)	0.25 (0.18–0.33)	0.22 (0.16–0.32)

ARDS = acute respiratory distress syndrome, CARDS = coronavirus disease 2019-associated acute respiratory distress syndrome, ECMO = extracorporeal membrane oxygenation, IQR = interquartile range, ICU = intensive care unit, pDDI = potential drug–drug interaction, D = clinically relevant interaction that is best avoided, C = clinically relevant interaction that can be handled, B = clinical outcome of the interaction is uncertain and/or may vary; * *p* < 0.05; for non-COVID-19 vs. COVID-19-induced ARDS; for ECMO vs. no ECMO.

**Table 2:** Most frequently identified D-graded pDDIs.

Severity D-Graded pDDIs of Patients with ARDS (*n* = 189)					
Substance A	Substance B	Organ System	Potential Consequences	PD/PK	Frequency
Erythromycin	Midazolam	CNS	CNS depression	PK	37
Propofol	Vasopressin	CV	Risk of QTc prolongation	PD	28
Amiodarone	Propofol	CV	Risk of QTc prolongation	PD	26
Midazolam	Voriconazole	CNS	CNS depression	PK	22
Fluconazole	Propofol	CV	Risk of QTc prolongation	PD	16
Amiodarone	Vasopressin	CV	Risk of QTc prolongation	PD	11
Dobutamine	Vasopressin	CV	Risk of QTc prolongation	PD	11
Linezolid	Piritramide	General	Serotonergic effects	PD	10
Erythromycin	Voriconazole	CV	Cardiac arrest	PK	8
Fluconazole	Midazolam	CNS	CNS depression	PK	7

ARDS = acute respiratory distress syndrome, CNS = central nervous system, CV = cardiovascular, PD = pharmacodynamics, D = clinically relevant interaction that is best avoided, pDDI = potential drug–drug interaction, PK = pharmacokinetics.

**Table 3:** Most frequent drug pairs with ATC codes of D-graded pDDIs.

ATC Code for Substances		Frequency Non-CARDS	Frequency CARDS	Frequency No ECMO (Both ARDS Conditions)	Frequency ECMO (Both ARDS Conditions)
Anti-infectives for systemic use (J)	Nervous system (N)	71	60	45	86
Cardiovascular system (C)	Nervous system (N)	24	20	14	31
Systemic hormonal preparations (H)	Nervous system (N)	22	8	13	17
Cardiovascular system (C)	Systemic hormonal preparations (H)	15	10	5	19
Cardiovascular system (C)	Anti-infectives for systemic use (J)	13	9	6	16
Systemic hormonal preparations (H)	Anti-infectives for systemic use (J)	13	2	4	11
Anti-infectives for systemic use (J)	Anti-infectives for systemic use (J)	13	7	8	12

ARDS = acute respiratory distress syndrome, ATC = Anatomical Therapeutic Chemical, CARDS = coronavirus disease 2019-associated acute respiratory distress syndrome, D = clinically relevant interaction that is best avoided, ECMO = extracorporeal membrane oxygenation, pDDI = potential drug–drug interaction.

**Table 4:** Differences in pDDIs between the two different analytic methods.

	Drug Elimination Analysis			24 h Analysis		
ID	Substance A	Substance B	PD/PK	Substance A	Substance B	PD/PK
2	Clarithromycin	Hydrocortisone	PK	Nebivolol	Urapidil	PD
				Butyl scopolamine	Metoclopramide	PD
5	Amiodarone	Levofloxacin	PD	Canrenoate	Potassium	PD
	Amiodarone	Quetiapine	PD			
	Amiodarone	Trazodone	PD			
	Amiodarone	Metoclopramide	PD			
6	Amiodarone	Erythromycin	PK	Propofol	Erythromycin	PD
	Amiodarone	Propofol	PD			
8	Erythromycin	Propofol	PD	Canrenoate	Potassium	PD
	Erythromycin	Prednisolone	PK			
9	Trimethoprim	Torasemide	PD	Potassium	Trimethoprim	PD
10	Azithromycin	Propofol	PD	Midazolam	Propofol	PD
	Enoxaparin	Metamizole	PD	Ondansetron	Propofol	PD
	Heparin	Metamizole	PD			
	Ondansetron	Paracetamol	PK			
	Pantoprazole	Quetiapine	PK			
12	Cisatracurium	Rocuronium	PD			
	Enoxaparin	Metamizole	PD			
	Lorazepam	Quetiapine	PK			
13	Enoxaparin	Metamizole	PD			
15				Nitroglycerin	Heparin	PD
17	Amiodarone	Naloxegol	PK	Canrenoate	Potassium	PD
	Dexamethasone	Naloxegol	PK			
	Isavuconazole	Voriconazole	PK			
18				Furosemide	ASA	PD

ASA = acetylsalicylic acid, PK = pharmacokinetic, PD = pharmacodynamic.

## Data Availability

The data presented in this study are available upon request from the corresponding author. The data are not publicly available due to data protection.

## Supplementary Materials

The following supporting information can be downloaded at: https://www.mdpi.com/article/10.3390/pharmaceutics16030303/s1, Table S1: Most frequently identified D-graded pDDIs; Table S2: Demographics and pDDIs of patients in the subgroup analysis. Table S3: Detailed differences in pDDIs between the two different analytic methods.

## Abbreviations

ATC	Anatomical Therapeutic Chemical
ARDS	Acute respiratory distress syndrome
CARDS	Coronavirus disease 2019-associated acute respiratory distress syndrome
COVID-19	Coronavirus disease 2019
DDI	Drug–drug interaction
ECMO	Extracorporeal membrane oxygenation
ICU	Intensive care unit
IQR	Interquartile range
PD	Pharmacodynamic
pDDI	Potential drug–drug interaction
PK	Pharmacokinetic
SARS-CoV-2	Severe acute respiratory distress syndrome coronavirus disease 2
SmPC	Summary of Product Characteristics
