# Supplementary material for: Drug–Drug Interactions in Patients with Acute Respiratory Distress Syndrome
Source: Pharmaceutics. 2024 Feb 21;16(3):303. doi: 10.3390/pharmaceutics16030303 (PMC10974914; doi:10.3390/pharmaceutics16030303)
Supplement: Supplementary file 1 [file pharmaceutics-16-00303-s001.zip › pharmaceutics-2868304-supplementary.pdf]

pharmaceutics

Article

Drug–Drug Interactions in Patients with Acute Respiratory Distress Syndrome

Thorsten Bischof [1], Christoph Schaller [1], Nina Buchtele [2], Thomas Staudinger [2], Roman Ullrich [3], Felix Kraft [3], Marine L. Andersson [4], Bernd Jilma [1] and Christian Schoergenhofer [1,*]

1 Department of Clinical Pharmacology, Medical University of Vienna, 1090 Vienna, Austria; thorsten.bischof@meduniwien.ac.at (T.B.); bernd.jilma@meduniwien.ac.at (B.J.)
2 Intensive Care Unit 13i2, Department of Medicine 1, Medical University of Vienna, 1090 Vienna, Austria; nina.buchtele@meduniwien.ac.at (N.B.); thomas.staudinger@meduniwien.ac.at (T.S.)
3 Department of Anesthesiology, Medical University of Vienna, 1090 Vienna, Austria; roman.ullrich@meduniwien.ac.at (R.U.); felix.kraft@meduniwien.ac.at (F.K.)
4 Division of Clinical Pharmacology, Department of Laboratory Medicine, Karolinska Institutet, Karolinska University Hospital, 141 86 Stockholm, Sweden; marine.andersson@regionstockholm.se
* Correspondence: christian.schoergenhofer@meduniwien.ac.at; Tel.: +43-1-40400-29810

Table S1. Most frequently identified D-pDDIs.

Substance A	Substance B	Organ system	potential consequences	PD/PK	frequency
**Severity D-pDDIs of non-CARDS patients (n=100)**					
Propofol	Vasopressin	CV	risk of QTc prolongation	PD	21
Amiodarone	Propofol	CV	risk of QTc prolongation	PD	13
Fluconazole	Propofol	CV	risk of QTc prolongation	PD	11
Erythromycin	Midazolam	CNS	CNS depression	PK	11
Midazolam	Voriconazole	CNS	CNS depression	PK	9
Amiodarone	Vasopressin	CV	risk of QTc prolongation	PD	7
Linezolid	Piritramide	general	serotonergic effects	PD	7
Fluconazole	Midazolam	CNS	CNS depression	PK	7
Dobutamine	Vasopressin	CV	risk of QTc prolongation	PD	6
**Severity D-pDDIs of CARDS patients (n=89)**					
Erythromycin	Midazolam	CNS	CNS depression	PK	26
Amiodarone	Propofol	CV	risk of QTc prolongation	PD	13
Midazolam	Voriconazole	CNS	CNS depression	PK	13
Propofol	Vasopressin	CV	risk of QTc prolongation	PD	7
Erythromycin	Voriconazole	CV	cardiac arrest	PK	6
Fluconazole	Propofol	CV	risk of QTc prolongation	PD	5
Amiodarone	Vasopressin	CV	risk of QTc prolongation	PD	4
Dobutamine	Vasopressin	CV	risk of QTc prolongation	PD	4
Linezolid	Piritramide	general	serotonergic effects	PD	4
**Severity D-pDDIs of no-ECMO treatment (both ARDS conditions) (n=85)**					
Propofol	Vasopressin	CV	risk of QTc prolongation	PD	12
Erythromycin	Midazolam	CNS	CNS depression	PK	11
Amiodarone	Propofol	CV	risk of QTc prolongation	PD	8
Fluconazole	Propofol	CV	risk of QTc prolongation	PD	6
Midazolam	Voriconazole	CNS	CNS depression	PK	5
Citalopram	Levofloxacin	CV	risk of QTc prolongation	PD	3
Linezolid	Piritramide	general	serotonergic effects	PD	3
Fluconazole	Midazolam	CNS	CNS depression	PK	3
Propofol	Sotalol	CV	risk of QTc prolongation	PD	3
**Severity D-pDDIs of ECMO treatment (both ARDS conditions) n=104**					
Erythromycin	Midazolam	CNS	CNS depression	PK	26
Amiodarone	Propofol	CV	risk of QTc prolongation	PD	18
Midazolam	Voriconazole	CNS	CNS depression	PK	17
Propofol	Vasopressin	CV	risk of QTc prolongation	PD	16
Fluconazole	Propofol	CV	risk of QTc prolongation	PD	10
Amiodarone	Vasopressin	CV	risk of QTc prolongation	PD	9
Dobutamine	Vasopressin	CV	risk of QTc prolongation	PD	9
Linezolid	Piritramide	general	serotonergic effects	PD	7
Erythromycin	Voriconazole	CV	cardiac arrest	PK	6

ARDS = acute respiratory distress syndrome, CARDS = coronavirus disease-2019 associated acute respiratory distress syndrome, CNS = central nervous system, CV = cardiovascular, PK = pharmacokinetic, PD = pharmacodynamic, ECMO = extracorporeal membrane oxygenation, pDDI = potential drug-drug-interaction, D = clinically relevant interaction that is best avoided

Table S2. Demographics and pDDIs of patients in the subgroup analysis.

	Overall	non-CARDS	CARDS	no ECMO (both ARDS conditions)	ECMO (both ARDS conditions)
**included patients, n**	20 (100)	10 (100)	10 (100)	8 (100)	12 (100)
**Sex, female (%)**	5 (25)	4 (40)	1 (10)	2 (25)	3 (25)
**Age, median (IQR)**	56 (46-61)	56 (54-61)	56 (34-62)	58 (33-63)	56 (53-61)
**ICU days, median (IQR)**	22 (16-24)	17 (15-22)	24 (21-26)	19 (16-26)	22 (16-24)

ICU = intensive care unit, ARDS = acute respiratory distress syndrome, CARDS = coronavirus disease-2019 associated acute respiratory distress syndrome, ECMO = extracorporeal membrane oxygenation, pDDI = potential drug-drug-interaction

Table S3. Detailed differences in pDDIs between the two different analytic methods

ID	pDDI severity	Substance A	Substance B	Organ system	potential consequences	PD/PK
			**Drug elimination analysis**			
2	C	Hydrocortisone	Clarithromycin	PK	increased hydrocortisone concentration	PK
5	D	Amiodaron	Levofloxacin	CV	risk of QTc prolongation	PD
5	C	Amiodaron	Quetiapine	CV	risk of QTc prolongation	PD
5	B	Amiodaron	Trazodone	CV	risk of QTc prolongation	PD
5	C	Amiodaron	Metronidazole	CV	risk of QTc prolongation	PD
6	D	Amiodaron	Erythromycin	PK	increased amiodaron concentration	PK
6	D	Amiodaron	Propofol	CV	risk of QTc prolongation	PD
8	C	Erythromycin	Propofol	CV	risk of QTc prolongation	PD
8	B	Erythromycin	Prednisolone	PK	increased prednisolon concentration	PK
9	B	Trimethoprim	Torsemide	electrolytes	hyponatremia	PD
10	C	Azithromycin	Propofol	CV	risk of QTc prolongation	PD
10	B	Enoxaparin	Metamizole	CV	increased risk of bleeding	PD
10	B	Heparin	Metamizole	CV	increased risk of bleeding	PD
10	B	Ondansetron	Paracetamol	PK	reduced paracetamol effect	PK
10	B	Pantoprazole	Quetiapine	PK	increased quetiapine concentration	PK
12	B	Cisatracurium	Rocuronium	musculo-skeletal	neuromuscular blocking	PD
12	B	Enoxaparin	Metamizole	CV	bleeding	PD
12	B	Lorazepam	Quetiapine	PK	increased quetiapine concentration	PK
13	B	Enoxaparin	Metamizole	CV	bleeding	PD
17	B	Amiodaron	Naloxegol	PK	increased naloxegol concentration	PK
17	B	Dexamethasone	Naloxegol	PK	decreased naloxegol concentration	PK
17	C	Isavuconazole	Voriconazole	PK	increased isavuconazole concentration	PK
			**24-hour analysis**			
2	C	Urapidil	Nebivolol	CV	hypotension	PD
2	B	Butyl scopolamine	Metoclopramide	GI	decreased propulsive effect	PD
5	C	Canrenoate	Potassium	electrolytes	hyperkalemia	PD
6	C	Propofol	Erythromycin	CV	QTc	PD
8	C	Canrenoate	Potassium	electrolytes	hyperkalemia	PD
9	C	Potassium	Trimethoprim	electrolytes	hyperkalemia	PD
10	B	Midazolam	Propofol	CNS	CNS depression	PD
10	B	Propofol	Ondansetron	CV	risk of QTc prolongation	PD
15	C	Nitroglycerin	Heparin	general	resistance to heparin	PD
17	C	Canrenoate	Potassium	electrolytes	hyperkalemia	PD
18	B	Furosemide	ASA	renal	renal failure	PD

ASA = acetylsalicylic acid, PK = pharmacokinetic, PD = pharmacodynamic, pDDI = potential drug-drug-interaction, D = clinically relevant interaction that is best avoided, C = clinically relevant interaction that can be handled, B = clinical outcome of the interaction is uncertain and/or may vary
